# Wnt3a promotes epithelial–mesenchymal transition, migration, and proliferation of lens epithelial cells

**Published:** 2012-07-18

**Authors:** Xiu-li Bao, Hui Song, Zhuo Chen, Xin Tang

**Affiliations:** Clinical college of ophthalmology, Tianjin Medical University, Tianjin Eye Hospital, Tianjin, China

## Abstract

**Purpose:**

Posterior capsular opacification (PCO) is caused mainly by the epithelial–mesenchymal transition (EMT), proliferation, and migration of human lens epithelial (HLE) cells. wingless (Wnt) signaling has been implicated in the fibrotic process by inducing EMT and increasing the proliferation of epithelial cells. This study investigated the role of Wnt3a in PCO formation.

**Methods:**

Wnt3a was overexpressed in the HLE B-3 cell line by transfected Wnt3a-pcDNA3 plasmid. The expressions of Wnt/β-catenin signaling component proteins, including β-catenin, E-cadherin, fibronectin, c-Myc, and cyclin D1, were detected by western blot analysis and immunocytofluorescence to confirm the efficiency of transfection efficiency and analyze the effects of overexpression. HLE migration ability was evaluated by transwell migration and wound healing assays, whereas HLE proliferation was analyzed by MTT [3-(4,5-dimethylthiazol-2-yl) −2,5-diphenyltetrazolium bromide] assay and flow cytometry.

**Results:**

Overexpression of Wnt3a resulted in upregulated expression of β-catenin, c-Myc, and cyclin D1. Expression of the lens epithelial marker E-cadherin was down-regulated in Wnt3a-overexpressing HLE B-3 cells, whereas that of the mesenchymal marker fibronectin was upregulated. In addition, the morphology of HLE B-3 cells changed from the classic spindle shape to an irregular form. Overexpression of Wnt3a could enhance the ability of migration as determined by transwell migration and wound healing assays as well as promoted the proliferation of HLE B-3 cells by MTT assay and flow cytometry analysis.

**Conclusions:**

Wnt3a can induce EMT, migration, and proliferation of HLE cells and may be a valuable therapeutic target for the prevention and treatment of PCO.

## Introduction

Posterior capsular opacification (PCO), also known as secondary cataract, is a common long-term complication of modern cataract surgery. It has an incidence ranging from 20% to 40% of patients 2–5 years after surgery [1]. It is mainly caused by secondary pathological progression of postoperative residual lens epithelial cells, including proliferation, migration, epithelial–mesenchymal transition (EMT), collagen deposition, and lens fiber regeneration [1,2]. Cataract surgery has been demonstrated to induce a wound-healing and fibrogenic response in the lens, with the leftover lens epithelial cells undergoing EMT and bearing morphological and molecular resemblance to fibrotic lesions in PCO [3,4]. Morphologically, the EMT of lens epithelial cells contributes to the regeneration of crystallin-expressing lenticular fibers and formation of Elschnig’s pearls and Soemmering’s ring [2,4,5]. Furthermore, EMT results in folds and wrinkles of lens epithelial cells on the posterior capsule [1]. Several studies have suggested that some important regulators of EMT not only play a role in the transformation of lens epithelial cells but also influence the healing process after cataract surgery [6,7].

EMT is the process through which epithelial cells change their phenotype, acquire mesenchymal properties, and possibly increase their capability to migrate and/or synthesize interstitial matrices [8]. Molecular hallmarks of EMT include down-regulation of E-cadherin, which is responsible for the loss of cell–cell adhesion; upregulation of matrix-degrading proteases and mesenchymal-related proteins, such as vimentin and fibronectin; reorganization of the actin cytoskeleton to activate the motility machinery; and nuclear translocation of transcription factors. EMT is possibly involved in the pathogenesis of fibrotic disorders in the kidney, lung, liver, eye, and serosal membranes [8,9]. Recent research has shown that the wingless (Wnt)/β-catenin pathway is one of the main molecular pathways involved in the induction of EMT during the fibrogenic process [8].

The Wnt family of secreted signaling proteins plays an essential role in organogenesis, tissue homeostasis, and tumor formation [10]. Wnt signals are implicated in extensive activities, ranging from mitogenic stimulation to differentiation, changes in polarity, and differential cell adhesion [11]. Activation of Wnt signaling leads to β-catenin nuclear translocation and complex formation with lymphoid enhancer-binding factor/T cell factor ((LEF/TCF) transcription factors, followed by transcriptional activation of target genes in the nucleus [11]. The Wnt/β-catenin signaling pathway, also known as the canonical Wnt pathway, involves physiologic and pathophysiological processes, including cell proliferation, differentiation, and migration [12]. An overactive Wnt signaling pathway leads to a variety of abnormalities and diseases. Recently, several studies have shown the involvement of the Wnt pathway in EMT and fibrosis of epithelial cells [13,14]. Wnt3a, a prominent member of the Wnt family, can induce the accumulation of β-catenin and activation of the canonical Wnt signaling pathway [15]. Chong et al. [16] reported that transforming growth factor β (TGFβ) induces the EMT of lens epithelial cells and promotes Wnt expression during cataract development and that Wnt signaling is involved in EMT and the development of fibrotic plaques in the lens in vitro and in vivo. In spite of these findings, little is known about the role of Wnt3a in PCO.

We hypothesized that regulation of PCO and the EMT of lens epithelial cells could be attributed to functional aberrations in Wnt signaling. In this study, we explored the role of Wnt3a in PCO based on the overexpression of Wnt3a.

## Methods

### Cell culture

The immortalized human lens epithelial (HLE) cell line HLE B-3 was obtained from American Type Culture Collection (Manassas, VA). HLE B-3 was maintained in Eagle’s minimum essential medium plus 20% heat-inactivated fetal bovine serum, 100 U/ml of penicillin G, and 100 μg/ml of streptomycin in humidified 5% CO_2_ at 37 °C.

### Plasmid construction and cell transfection

The fragment of *Wnt3a* cDNA containing full-length cDNA of the human *Wnt3a* gene was obtained by PCR using a *Wnt3a* cDNA library (kindly provided by Dr. Tang Hua, Tianjin Life Science Research Center and Basic Medical School, Tianjin Medical University, Tianjin, China) as template. The forward primer 5ʹ-GAG GAA TTC GCC ACC ATG GCC CCA CTC GGA TAC-3ʹ and reverse primer 5ʹ-GAC AGC TCG AGG CCT TGC AGG TGT GCA CGT CGT AG-3ʹ were used. The PCR conditions were as follows: 95 °C for 4 min; 35 cycles of 94 °C for 1 min, 68 °C for 90 s, and 72 °C for 3 min; and 72 °C for 10 min. After digestion by EcoRI and XhoI, the PCR product was subcloned into pcDNA3 vector, which was termed Wnt3a-pcDNA3. The resulting constructs were confirmed by DNA sequencing. The pcDNA3 empty plasmid was used as negative control.

HLE B-3 cells were transfected with Wnt3a-pcDNA3 plasmid or pcDNA3 plasmid using Lipofectamine 2000 Transfection Reagent (Invitrogen, Carlsbad, CA) according to the manufacturer’s instructions. The transfection efficacy of HLE B-3 cells was 87%. HLE B-3 cells were seeded either in a 24-well plate (5×10^5^ cells/well) or in a 25 ml culture flask (1.5×10^5^ cells). After 16 h at approximately 60% confluence, the cells were transfected with Wnt3a-pcDNA3 plasmid (1 μg/well, 4 μg/flask) in 1 or 10 μl of transfection reagent in a final volume of 500 μl or 1 ml of transfection medium (GIBCO BRL, Grand Island, NY). Four hours after transfection, full culture medium without antibiotics was added to the mixture.

### Western blot analysis

After 48 h of transfection, HLE B-3 cells were lysed in ice-cold RIPA buffer containing 1% protease inhibitors (Roche, Basel, Switzerland). Proteins were harvested, and protein concentration was determined using BCA assay (Pierce, Rockford, IL). Proteins (30 μg per lane) were separated by SDS–PAGE and transferred to a nitrocellulose membrane (Millipore, Bedford, MA). After blocking with 5% nonfat dry milk in TBS containing Tween-20 for 2 h at room temperature, membranes were incubated overnight with rabbit anti-human E-cadherin, fibronectin, β-catenin, c-Myc, cyclin D1, or actin primary antibodies (Abcam Biotechnology, Cambridge, UK) at 4 °C, diluted 1:1,000 in 0.5% nonfat dry milk in TBS. Nitrocellulose membranes were washed three times in TBS containing 0.1% Tween-20 and incubated with goat anti-mouse IgG HRP (1:5,000; Santa Cruz, Santa Cruz, CA) or goat anti-rabbit IgG HRP (1:5,000; Santa Cruz) for 90 min at room temperature. After three washes in TBS and two washes in PBS, specific bands were detected using enhanced chemiluminescence reagent (Millipore, Billerica, MA) and by exposure to chemiluminescence film.

### Immunocytofluorescence staining

Cells grown on coverslips were fixed with 10% formalin at −20 °C for 10 min and then quenched with 50 mM NH_4_Cl for 10 min, followed by 0.2% Triton for 10 min. Cells were then blocked with 3% BSA for 1 h and incubated with primary antibodies (E-cadherin, 1:50; fibronectin, 1:50; β-catenin, 1:50; Abcam Biotechnology) overnight at 4 °C. After washing with PBS, cells were incubated with fluorophore-labeled secondary antibodies (Invitrogen, Carlsbad, CA) for 1 h. The cells were mounted with mounting medium containing 4ʹ,6-diamino-2-phenylindole (DAPI; 1 μg/ml; Invitrogen). To visualize fluorescence, we used an epi-illumination fluorescence light microscope (BX50; Olympus, Inc., Tokyo, Japan) and a high-sensitivity cooled CCD camera (DP30BW; Olympus).

### MTT assay

An HLE B-3 cell suspension of 200 μl at the concentration of 1×10^4^ cells/ml was seeded on 96-well plates and transfected with Wnt3a vector or pcDNA3-HA as control. After 24, 48, 72, 96, and 120 h, 10 μl of MTT [3-(4,5-dimethylthiazol-2-yl) −2,5-diphenyltetrazolium bromide] (Sigma, St. Louis, MO) at 5 mg/ml was added to each well, and the plate was incubated at 37 °C for 4 h. The medium was then removed, and 100 μl of dimethyl sulfoxide was added to the wells. Insoluble formazan crystals were dissolved in dimethyl sulfoxide after 1 min of incubation with shaking, and absorbance was read at 490 nm on a microplate reader (Bio-tek Instruments, Winooski, VT).

### Flow cytometry analysis

After 72 h of transfection, HLE B-3 cells were washed with PBS, harvested and fixed with 95% ice-cold alcohol at 4 °C overnight, resuspended in 500 μl of propidium iodine (Sigma), and then incubated at 37 °C for 30 min in the dark. Stained cells were analyzed using a FACScan flow cytometer and Cell Quest analysis software (Becton Dickinson, San Jose, CA). Flow cytometry analysis was repeated three times.

### Transwell migration assay

HLE B-3 cell migration assay was performed using transwell cell culture inserts (Transwell Assay System; Corning, High Wyombe, UK) for 24-well plates. Two hundred microliters of serum-starved HLE B-3 cells (5×10^5^ cells/ml) was added to the upper polycarbonate membrane insert (pore size, 8 μm) and incubated at 37 °C in 5% CO_2_, whereas 300 μl of cultured medium with 20% fetal bovine serum was added to the lower chamber. After 48 h of incubation, the membranes were fixed with 5% glutaraldehyde in PBS for 10 min at room temperature and then stained with 0.5% toluidine blue in 2% Na_2_CO_3_ for 15 min. The number of migratory cells was counted five times in random fields of microscope. Experiments were performed in triplicate.

### Wound healing assay

HLE B-3 cells were seeded onto 35 mm dishes. After 24 h of transfection with the Wnt3a-pcDNA3 or pcDNA3-HA plasmid, the cells formed a fluent monolayer. A linear scratch was formed using a 10 μl pipette tip. Wounded monolayers were washed with PBS to remove detached cells and debris, and the complete medium was then replaced by Opti-DMEM. The ability of HLE B-3 cells to close the wounded space was used to assess their migration ability. The width of the “wound” obtained by light microscopy was recorded at 0, 12, 24, and 48 h. The results were analyzed three times.

### Statistical analysis

Data were analyzed using two-tailed Student’s *t*-test. p<0.05 was considered statistically significant.

## Results

### Overexpression of Wnt3a activated the Wnt/β-catenin signaling pathway

**Figure 1:**
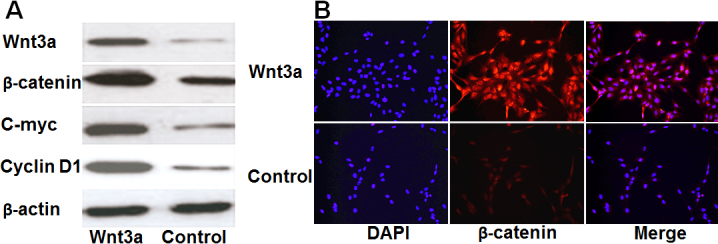
Wnt3a activates the Wnt/β-catenin signaling pathway in HLE B-3 cells. **A**: western blot analysis showed that overexpression of Wnt3a results in the upregulated expression of Wnt/β-catenin signaling component proteins, including β-catenin, c-Myc, and cyclin D1. β-Actin was used as internal control. **B**: Immunocytofluorescence also demonstrated increased expression of β-catenin protein (red) in Wnt3a-overexpressing cells compared with control cells. Nuclei were stained with DAPI (blue). Merged images are shown in the right panel.

Western blot analysis was used to confirm the transfection efficiency of Wnt3a. The results showed that the Wnt3a protein was significantly higher in transfected cells than in non-transfected cells. Wnt3a overexpression induced the upregulation of β-catenin, c-Myc, and cyclin D1 proteins, confirming that the Wnt/β-catenin signaling pathway was activated in HLE B-3 cells (Figure 1A). Immunocytofluorescence further confirmed the upregulation of β-catenin and nuclear translocation in Wnt3a-overexpressing cells compared with control cells (Figure 1B).

### Upregulation of Wnt3a induced the EMT of HLE B-3 cells

**Figure 2:**
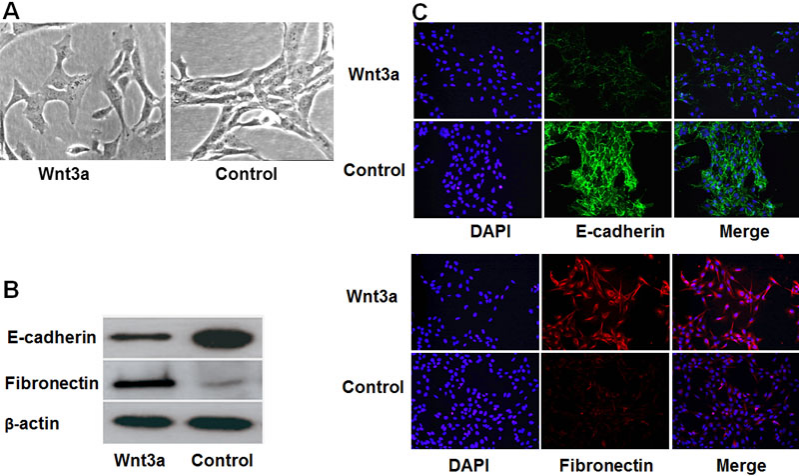
Wnt3a induces the EMT of HLE B-3 cells. **A**: Wnt3a-overexpressing cells had an irregular shape, whereas control cells had a spindle-shaped morphology (original magnification 400×). **B**: western blot analysis detected down-regulated epithelial protein E-cadherin in Wnt3a-overexpressing HLE B-3 cells compared with control cells. In contrast, the expression of mesenchymal protein fibronectin was upregulated in Wnt3a-overexpressing cells. **C**: Immunocytofluorescence showed that the expression of E-cadherin protein (green) was down-regulated in Wnt3a-overexpressing cells compared with control cells, whereas that of fibronectin (red) increased. DAPI (blue) was used for nuclear staining. Merged images are shown in the right panel.

The lens epithelial protein and mesenchymal protein were analyzed to validate the role of Wnt3a in the regulation of EMT. Western blot analysis found that overexpression of Wnt3a protein levels significantly down-regulated the epithelial phenotypic marker E-cadherin and upregulated the mesenchymal phenotypic marker fibronectin (Figure 2B). Immunocytofluorescence further confirmed that EMT was induced in HLE B-3 cells (Figure 2C). Moreover, compared with control cells, E-cadherin protein and fibronectin were clearly down-regulated and significantly upregulated in Wnt3a-overexpressing cells, respectively.

The morphology of HLE B-3 cells was examined 48 h post-transfection of Wnt3a plasmid. Their classic spindle-shaped morphology changed into an irregular form (Figure 2A). These findings suggested that Wnt3a-overexpressing cells achieved more mesenchymal characteristics.

### Upregulation of Wnt3a enhanced the migration of HLE B-3 cells

**Figure 3:**
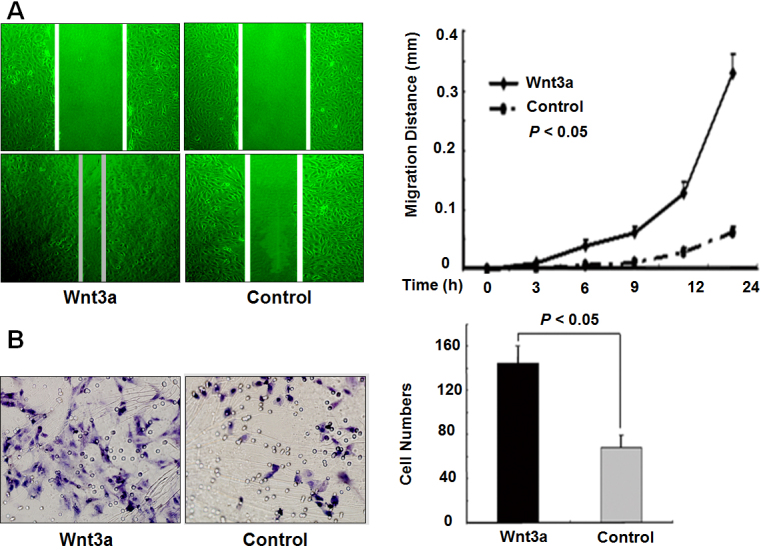
Wnt3a promotes the migration of HLE B-3 cells. **A**: Wound healing assay showed increased migration ability of HLE B-3 cells after 48 h of transfection with Wnt3a-pcDNA3 plasmid. The images were taken at 0 and 24 h after scratch was applied (left; original magnification 100×). The migration distance of the Wnt3a-overexpressing cells at different time points was significantly increased compared with that of control cells (right; p<0.05). **B**: Transwell migration assay. Cells on transwell inserts were stained (left; original magnification 200×). The number of migratory Wnt3a-overexpressing HLE B-3 cells was significantly higher than that of control cells (right; p<0.05).

Wound healing and transwell migration assays were performed and analyzed to investigate the effect of Wnt3a on cell migration. As shown in Figure 3A, Wnt3a-overexpressing cells moved faster compared with control cells. Wnt3a-overexpressing cells moved toward the gap and completed closure in less than 24 h, whereas control cells exhibited a delay in closure.

The migration ability of Wnt3a-overexpressing cells was unequivocally increased using transwell migration assay (Figure 3B). After 48 h of incubation, the number of migratory cells across the polycarbonate membrane in the Wnt3a-overexpressing group was higher than that in the control set (p<0.05). The results demonstrated that overexpression of Wnt3a establishes the ability of HLE B-3 cells to migrate.

### Upregulation of Wnt3a initiated the proliferation of HLE B-3 cells

**Figure 4:**
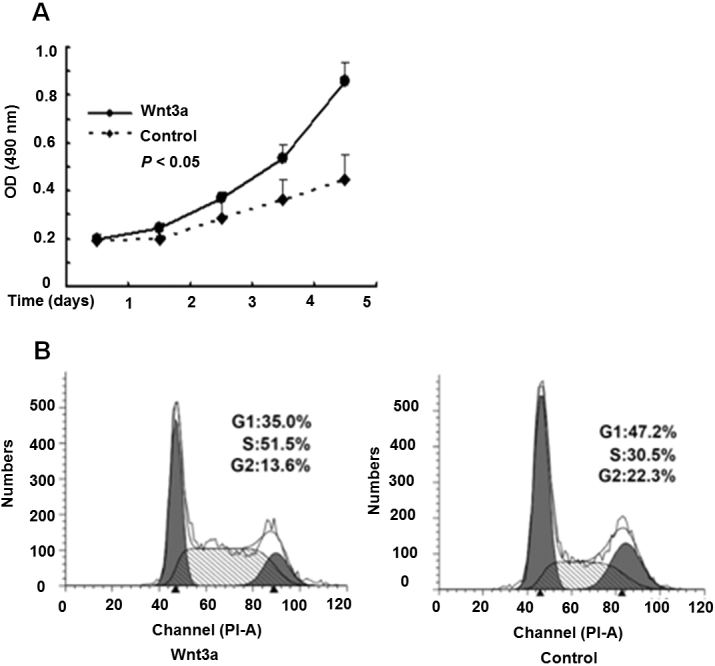
Wnt3a influences the proliferation and cell-cycle distribution of HLE cells. **A**: MTT assay showed that the OD at 490 nm values of Wnt3a-overexpressing cells increased compared with control cells, as determined every 24 h for 5 days (p<0.05). **B**: Representative flow cytometry analysis demonstrated that a higher percentage of Wnt3a-overexpressing HLE B-3 cells were in the S phase.

MTT assay was performed to examine the role of Wnt3a in the proliferation of HLE B-3 cells. The results revealed that Wnt3a-overexpressing HLE B-3 cells promoted HLE B-3 cell growth (Figure 4A). Consistently, flow cytometry analysis showed that the overexpression of Wnt3a increased the cell percentage of HLE B-3 cells in the S phase from 51.5% to 30.5% (p<0.05 versus control; Figure 4B).

## Discussion

PCO is a surgery-induced lens capsule repair process and fibrotic response characterized by remnant lens epithelial cell proliferation and migration, EMT, collagen deposition, and lens fiber regeneration [1]. The cells differentiated through EMT can change into myofibroblasts and fibroblasts, and this process can increase their capability to migrate and synthesize interstitial matrices [8,17]. The main molecular pathways involved in the induction of EMT include the Wnt/β-catenin, extracellular matrix/ILK, and TGFβ/Smad pathways [8]. We have demonstrated that Wnt3a could regulate EMT, migration, and proliferation of HLE cells.

Wnt signaling has been reported to contribute to lens fiber differentiation. Wnt3a induces lens differentiation by regulating accumulation of lens proteins through canonical Wnt signaling pathway. However, Wnt3a promotes the morphological aspects of fiber cell differentiation through β-catenin-independent Wnt signaling pathway related to FGF and BMP [18-20]. Non-canonical Wnt signaling pathway including Wnt/Planar Cell Polarity (PCP) signaling may be involved in regulating the complex cytoskeletal reorganization which is a key feature of EMT and fiber elongation in the lens [21]. How Wnt stimulation of epithelial to mesenchymal transition in the normal lens is not clear at present.

In this study, Wnt3a was used to examine the possible involvement of the canonical Wnt signaling pathway in regulating HLE cell behavior. Wnt3a signaling mainly activates this pathway [15], which is reliant on β-catenin signaling as a central signaling molecule. The Wnt/β-catenin pathway exerts remarkable control over cellular proliferation, differentiation, invasion, and adhesion [22,23]. The exact mechanism behind the ability of the Wnt/β-catenin pathway to induce EMT is not clear, but it fulfills two characteristics of β-catenin: as a structural protein in conjunction with E-cadherin that plays a role in cellular adhesion junctions and as an intermediary in the Wnt/β-catenin pathway. Loss of the E-cadherin/β-catenin adhesion complex triggers the adherens junctions to disassemble, consequently leading to cell migration [22], which is an important process in EMT [9,24]. Aberrant β-catenin signaling can occur in certain types of cancer and fibroproliferative disorders [25]. As for the second characteristic of β-catenin, much research has demonstrated that Wnt signaling through the frizzled receptor/LRP5/6 complex results in phosphorylation of Dishevelled, which in turn inactivates glycogen synthase kinase 3β. This inactivation allows β-catenin to translocate the nucleus and transactivate the Tcf/Lef transcription complex [15,25], which can induce transcription of cellular regulators of proliferation and differentiation, such as fibronectin [26], cyclin D1, and c-Myc [27,28]. The results of this study indicate that Wnt3a enhanced the expression of β-catenin and nuclear translocation of β-catenin, suggesting that Wnt3a signaling may activate the canonical pathway in HLE cells.

We also found that Wnt3a enhanced the expression of c-Myc and cyclin D1. c-Myc is not only a potential target of the Wnt/β-catenin signaling pathway but also a transcriptional regulator and a key component in stimulating cell-cycle progression, especially during the transition from the G_1_ phase to the S phase [29]. In addition, c-Myc seems to be active in a variety of tumors [30]. Cyclin D1 has a rate-limiting role in the G_1_–S phase transition and plays a critical role in normal and malignant cell growth [31]. These data suggest that c-Myc and cyclin D1 may be downstream effectors of Wnt3a in the Wnt/β-catenin signaling pathway in HLE cells.

EMT is essential to the developmental process of fibrotic diseases and cancer metastasis. EMT and ectopic proliferation of HLE cells have been suggested to contribute to the development of PCO [32]. E-cadherin forms part of tight junctional complexes, and its down-regulation in EMT accounts for the loss of cell–cell adhesion; moreover, the transepithelial barrier function is significantly altered in the fibrotic process [33]. Loss of E-cadherin in EMT has also been demonstrated in other organic diseases, such as those in the kidney, lung, and liver [34-36]. Fibronectin is a major mesenchymal marker, and it has been shown to participate in the conversion of various cell types into myofibroblasts under pathological conditions [37]. In this study, we have demonstrated that overexpression of Wnt3a resulted in down-regulation of E-cadherin and upregulation of fibronectin in HLE cells. Morphologically, the configuration of HLE cells changed from the classic spindle shape to an elongated fibroblastic form. Functionally, Wnt3a-overexpressing cells achieved increasing migration activity. These results showed that Wnt3a induced the EMT of lens epithelial cells.

Wnt3a has been reported to induce cell proliferation through β-catenin-dependent signaling pathways [38] or β-catenin-independent direct signaling [39]. We have shown that Wnt3a enhanced the proliferation of HLE cells, accompanied by increased expression of c-Myc and cyclin-D1. Cyclin D1 and c-Myc of the Wnt signal pathway targets were verified as β-catenin/Tcf4 pathway-dependent transcription genes. Nuclear translocation of β-catenin induced by Wnt3a has been suggested to directly induce c-Myc and cyclin D1 expression, thereby stimulating the proliferation of HLE cells.

In conclusion, this study suggests that Wnt3a is involved in the EMT, migration, and proliferation of HLE cells and may be a valuable therapeutic target for the prevention and treatment of PCO. However, the formation of PCO is a complicated process that is modulated by multiple signaling pathways. This fractional study was limited in that only one cell line (HLE B-3) was analyzed. Further study on accurate mechanisms and functions of Wnt3a in other cell lines and/or animals is clearly warranted.
